# MiR-4303 relieves chondrocyte inflammation by targeting ASPN in osteoarthritis

**DOI:** 10.1186/s13018-021-02731-9

**Published:** 2021-10-18

**Authors:** Chunyu Wang, Li Wang, Xingfa Guan, Changfeng Yue

**Affiliations:** 1 Department of Orthopedics, Chifeng Municipal Hospital, Chifeng, 024000 Inner Mongolia Autonomous Region China; 2 Department of Orthopedics, Second People’s Hospital of Rizhao City, Rizhao, 276808 Shandong Province China; 3 Department of Orthopedics, Huantai County People’s Hospital, Zibo, 256400 Shandong Province China; 4 Department of Orthopedics, Dongying District People’s Hospital, No. 96 Jinan Road, Dongying District, Dongying, 257000 Shandong Province China

**Keywords:** Osteoarthritis, miR-4303, ASNP, Chondrocyte inflammation

## Abstract

**Background:**

Osteoarthritis (OA) is a severe articular cartilage disease whose pathogenesis involves the inflammation of chondrocytes. MicroRNAs (miRNAs) are considered to be effective inflammation regulators. However, the regulatory mechanism of miRNAs in osteoarthritis needs to be further elucidated. In this paper, we aim to investigate the underlying mechanisms by which miR-4303 regulates osteoarthritis.

**Methods:**

RT-qPCR is performed to detect the mRNA expression levels of miR-4303, ASPN, PDIA3, PIK3CA, and TRAF3. CCK-8 assay and EdU assay are carried to assess chondrocyte viability. The protein expression levels of ASPN, PCNA, Ki-67, CyclinA1, CyclinB1, CyclinD2, p27, Bax, Bcl-2, cleaved caspase-3, and Cleaved caspase-9 were measured by western blot. FACs is performed to detect the cell cycle and apoptosis of chondrocyte. ELISA is conducted to assess the levels of TNF-β, IL-1β and IL-6 in the supernatant of chondrocytes. The potential binding sites of miR-4303 and ASPN are predicted by the miRDB database and confirmed by the dual-luciferase reporter gene assay.

**Results:**

Our findings illustrated that miR-4303 was down-regulated in arthritic tissues and LPS-induced chondrocytes; miR-4303 overexpression rescued the decrease in cell viability, cell cycle arrest and apoptosis induced by LPS. Furthermore, miR-4303 overexpression inhibited the release of inflammatory factors in LPS-induced chondrocytes, miR-4303 relieved chondrocyte inflammation via targeting ASPN.

**Conclusion:**

MiR-4303 serves as a prognostic biomarker and relieves chondrocyte inflammation via targeting ASPN. Our findings provide novel prognostic biomarkers in predicting the progression and prognosis of osteoarthritis.

## Introduction

Osteoarthritis (OA), the typical central form of arthritis, is accompanied by inflammation of chondrocytes [1]. Increasing evidence shows that the inflammatory mechanism mediates chondrocytes' biomechanical disorder and participates in the regulation of osteoarthritis progression [2]. Furthermore, many inflammatory mediators are released from chondrocytes following joint injury, which may become therapeutic targets for preventing or treating osteoarthritis [3]. Therefore, figuring out the inflammatory mechanism of chondrocytes will provide new treatment strategies for the treatment of osteoarthritis.

MiRNA is a series of non-coding RNA sequences of approximately 18–22 bp [4]. Reports show that miRNAs play essential roles in gene expression via regulating post-transcriptional translation, regulating various disease progression [5]. As expected, a variety of miRNAs are involved in the regulation of osteoarthritis progression. For example, miRNA-93 inhibits chondrocytes apoptosis through TLR4/NF-κB signaling pathway to relieve osteoarthritis [6]. In addition, Liu et al. reports show that chondrocyte proliferation is essential for the recovery of osteoarthritis, and miRNAs may regulate osteoarthritis progression by regulating chondrocyte bioactivity [7].

Furthermore, research has shown that miRNAs may relieve osteoarthritis by regulating the release of inflammatory cytokines in chondrocytes [8–11]. However, up to now, reports about the mechanism of miR-4303 have remained scanty. Therefore, we would try to explore the mechanism of miR-4303 in regulating chondrocyte bioactivity and chondrocyte inflammation.

Asporin (ASPN) belonged to the leucine-rich small proteoglycan (SLRP) family. Because of the unique D repeat sequence of aspartic acid residue at the N terminal of ASPN, it is considered a vital protein secreted by chondrocytes and participates in regulating osteoarthritis pathogenesis [12]. In addition, research has shown that ASPN regulates multiple cell proliferation, invasion and apoptosis [13]. What is more, ASPN regulates various cytokines to relieve osteoarthritis, such as TGF-β [14]. Thus, we speculated that ASPN might alleviate osteoarthritis by regulating chondrocyte bioactivity and releasing inflammatory cytokines.

In summary, ASPN alleviated osteoarthritis by regulating chondrocyte bioactivity and the release of inflammatory factors from chondrocytes. Therefore, ASPN was a vital regulator of osteoarthritis. This article aimed to explore the role of miR-4303 and ASPN in chondrocyte inflammation and its underlying mechanisms. Our research might contribute to providing new treatment strategies for the treatment of osteoarthritis.

## Materials and methods

### Patients and tissue specimen

Osteoarthritis tissues and corresponding normal tissues were obtained from surgically removed patients with pathological osteoarthritis (20–55 years old; 13 males and 11 females; *n* = 8) of The First Affiliated Hospital, College of Medicine, Zhejiang University between May 2018 and May 2019. These patients are free from diseases such as infectious diseases and cancers. All patients were informed, and their written consents were obtained before inclusion. The Ethics Committee of the First Affiliated Hospital, College of Medicine, Zhejiang University approved all experimental protocols (Zhejiang, China; Ethical approval no, PRO20180916-R1), and experimental procedures were conducted Declaration of Helsinki Principles.

### Cell culture

Chondrocytes (CP-H107) were obtained from Punuosai Life Technology Co., Ltd. (Wuhan, China). Dulbecco’s modified Eagle’s medium (DMEM, Roche, Basel, Switzerland) supplemented with 10% fetal bovine serum (FBS) (Roche, Basel, Switzerland) and 1% penicillin–streptomycin solution (Solarbio, Beijing, China) was applied to cultured cells in a humid incubator containing 5% CO_2_ at 37 °C.

### Chondrocyte inflammatory model

A total of 1 × 10^5^ chondrocytes were treated with 10 μL LPS (100 ng/mL) for 24 h to induce the chondrocyte inflammation model in vitro. LPS-induced chondrocytes were applied to subsequent experiments.

### Cell transfection

PcDNA3.1-ASPN plasmids and its negative control pcDNA3.1-NC (synthesized by Sangon Biotech, Shanghai, China) were transfected in 6-well plates at 500 ng each well. The miR-4303 mimics (synthesized by Sangon Biotech, Shanghai, China) and their corresponding controls were transfected at 100 nM each well. All the transfection was performed using Lipofectamine™ 3000 Transfection Reagent (Takara, Kusatsu, Japan). 48-h post-transfection, chondrocytes were treated with LPS for subsequent experiments.

### RT-qPCR

TRIZOL reagent (Takara, Kusatsu, Japan) was applied to extracted total RNA from LPS-treated chondrocytes or osteoarthritic tissues. M-MLV Reverse Transcriptase (RNase H) kit (Takara, Kusatsu, Japan) was performed to synthesize cDNA. In addition, RT-qPCR was performed as previously described [15]. Primers applied to this research are shown in Table 1.

**Table 1:** Primer sequences

Primer name	(5′–3′) primer sequences
F-miR-4303	5′-AGAAAATAGCTTCTGAGC-3′
R-miR-4303	5′-ACCATGGTTAGCTCCTAAGCT‐3′
F-ASPN	5′-GGATTTTAAACGATACAAA-3′
R-ASPN	5′-GCCTTCAGTAAATGTTCATTA‐3′
F-PDIA3	5′-ATGGGCCTGTGA AGGTAGTGG-3′
R-PDIA3	5′-TGACCACACCAA GGGGCATA-3′
F-PIK3CA	5′-ATTTCATGAAACAAATGAATGATGCACA-3′
R-PIK3CA	5′-CCATTTTTGTTGTCCAGCCACCATGAC-3′
F-TRAF3	5′-GCGTCGACCATGGAGTCGAGTAAAAAGA-3′
R-TRAF3	5′-TTGCGGCCGCTCAGGGATCGGGCAGATCCG-3′
F-U6	5′- CTCGCTTCGGCAGCACATATACT ‐3′
R-U6	5′-ACGCTTCACGAATTTGCGTGTC‐3′
F-GAPDH	5′-GAGTCAACGGATTTGGTCGT‐3′
R-GAPDH	5′-TTGATTTTGGAGGGATCTCG‐3′

### CCK-8 assay

According to the instructions, chondrocyte cell viability was analyzed by Cell Counting Kit-8 (Beyotime, Nanjing, China). In detail, chondrocytes were cultured in a 96-well plate after corresponding treatment. A total of 10 μl CCK-8 solution was added to each well at indicated times. Subsequently, the medium mixed with CCK-8 solution was added to a new 96-well plate. The fluorescent microplate reader was carried to detect the absorbance, which reflected cell viability at 450 nm.

### EdU assay

Prepared EdU medium was added into LPS-treated chondrocytes 48-h post-transfection. Following incubation for 2 h, the medium was discarded; the cells were digested with trypsin and washed twice with 1 × PBS. After fixing with formaldehyde for 30 min, cells were decolorized with glycine, washed twice with 1 × PBS, twice with 1 × PBS containing 0.5% Triton X-100 for 10. Finally, staining was performed by cell-light™ EdU Cell Proliferation Detection (Sigma, St. Louis, MO, USA). For specific operations, please refer to the operating instructions.

### Western blot

Total proteins were isolated from LPS-treated chondrocytes transfected with corresponding plasmids using cell lysis buffer (Beyotime, Nanjing, China). Western blots were conducted according to previously described procedures [16]. All antibodies used in this research were obtained from Abcam (Cambridge, UK, 1:1000), including PCNA (ab29), Ki-67 (ab270650), CyclinA1 (ab53699), CyclinB1 (ab32053), CyclinD2 (ab207604), p27 (ab32034), Bax (ab32503), Bcl-2 (ab32124), Cleaved-casapase3 (ab32042) and Cleaved-casapase9 (ab2324). The optical density of protein bands was quantified by Image J software (ImageJ Software Inc., USA).

### FACs analysis

A total of 1 × 10^5^ chondrocytes were cultured in a 12-well plate using serum-free DMEM for 24 h, centrifuged at 1500 g for 5 min and washed with pre-cooled 1 × PBS twice. Subsequently, cells were fixed with 70% ethanol (Solarbio, Beijing, China), placed at 20 °C for 15 min, then centrifuged at 1500 g for 5 min, and washed twice with pre-chilled 1 × PBS. Subsequently, cells were incubated with 10 μg/μL DNase-free RNaseA (Sigma, St. Louis, USA) for 45 min at 37 °C to eliminate RNA, and washed twice with pre-chilled 1 × PBS. Finally, following centrifuging at 150*g* for 5 min, the cells were incubated with 1 mg/mL iodide (Sigma, St. Louis, USA) in the dark at 4 °C for 12 min. The percentage of cells at each cell cycle phase is quantified in a flow cytometer and analyzed by ModiFit software (Olympus, Tokyo, Japan). Besides, according to instructions, Annexin-FITC/PI Apoptosis Detection Kit (Beyotime, Nanjing, China) was combined with flow cytometry to detect cell apoptosis, according to manufacturer’s instructions [17]. In addition, modified software (Olympus, Tokyo, Japan) was performed to analyze the data.

### ELISA assay

After corresponding treatment, the supernatant of chondrocyte cells was harvested. The inflammatory factors' expression level, including TNF-α, IL-1β and IL-6, was determined using ELISA kits (Roche, Basel, Switzerland).

### Bioinformatics and double luciferase reporter assay

MiRDB was used to predict putative target genes. The luciferase vectors, including wild-type (AAGCUCUAUAUAAAUGCUCAGAG) or mutant (AAGCUCUAUAUAAAUCGAGUCUG) 3′-UTR of ASPN with the miR-4303 binding site, were purchased from Sangon Biotech (Shanghai, China). Wild-type or mutant 3′-UTR of ASPN were transfected at 500 ng/well. Subsequently, MiR-4303 mimics (100 nM) or NC mimics (100 nM) were co-transfected into chondrocytes using the Lipofectamine™ 3000 (Takara, Kusatsu, Japan) according to the manufacturer’s instructions. Luciferase activity was assessed by Dual-Light Chemiluminescent Reporter Gene Assay System (Applied Biosystems, Foster City, USA) and normalized to Renilla luciferase activity [18].

### Statistical analysis

The mean ± SD represented data from three independent experiments. GraphPad Prism version 5.0 software (GraphPad Software, Inc.) was used for statistical analysis of all data. Student’s *t* test or one-way ANOVA was used to compare two groups, and Tukey post-test was used for comparison within multiple groups. A *P* value of  < 0.05 indicated that the difference was statistically significant.

## Results

### MiR-4303 is down-regulated in arthritic tissues and LPS-induced chondrocytes

**Fig. 1:**
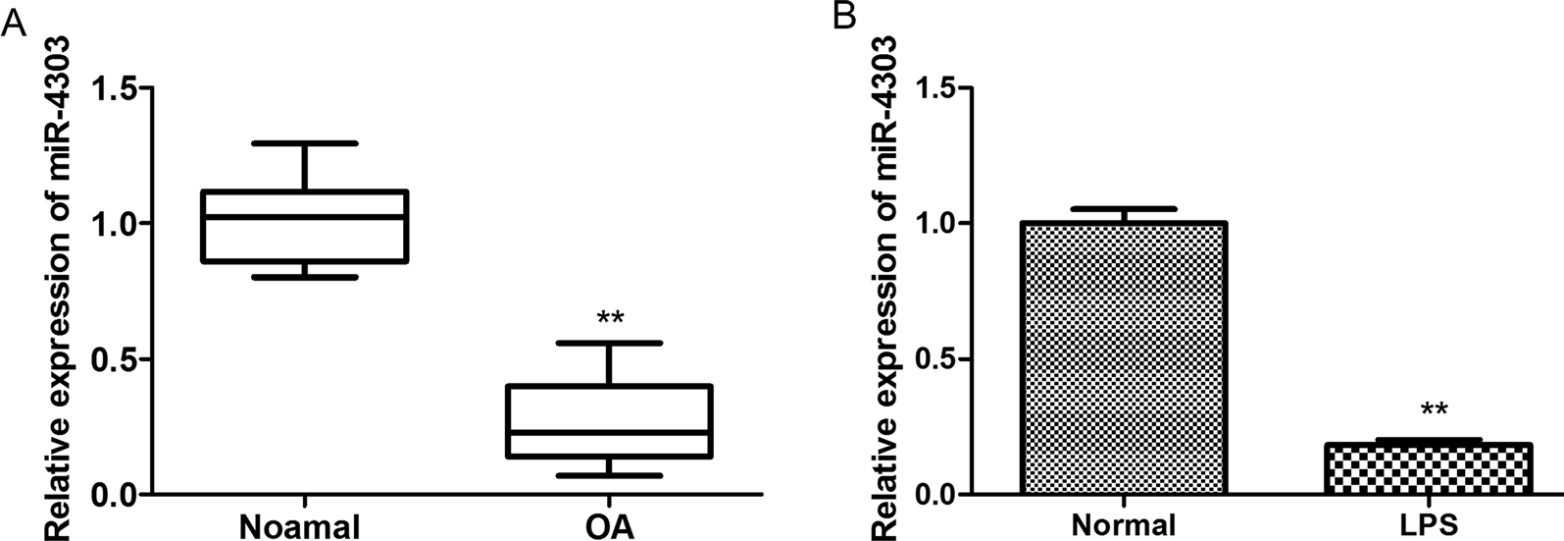
MiR-4303 was down-regulated in arthritic tissues and LPS-induced chondrocytes. **A** RT-qPCR was performed to assess the level of miR-4303 in arthritic tissues (*n* = 8), corresponding normal tissues (*n* = 8) served as the negative control for arthritic tissues. **B** The level of miR-4303 in LPS-treated CP-H107 cells (the concentration of LPS: 100 ng/mL). Student’s *t* test or one-way ANOVA was used for comparison between two groups. **P < 0.01 versus Normal group. Error bars represented SD. Data represent three independent experiments

It was reported that miRNAs were crucial regulators for diverse molecular and cellular activities, impacting numerous physiological and pathological processes [19]. Therefore, to explore the role of miR-4303 in arthritis, RT-qPCR was conducted to assess miR-4303 expression in arthritic tissues and LPS-induced chondrocytes. Results showed that miR-4303 was significantly down-regulated in arthritic tissues and LPS-induced chondrocytes, compared with control (*P* < 0.01; Fig. 1A, B), indicating miR-4303 played a negative role in chondrocyte inflammation.

### MiR-4303 overexpression rescued the decrease in cell viability, cell cycle arrest and promotion of apoptosis induced by LPS

**Fig. 2:**
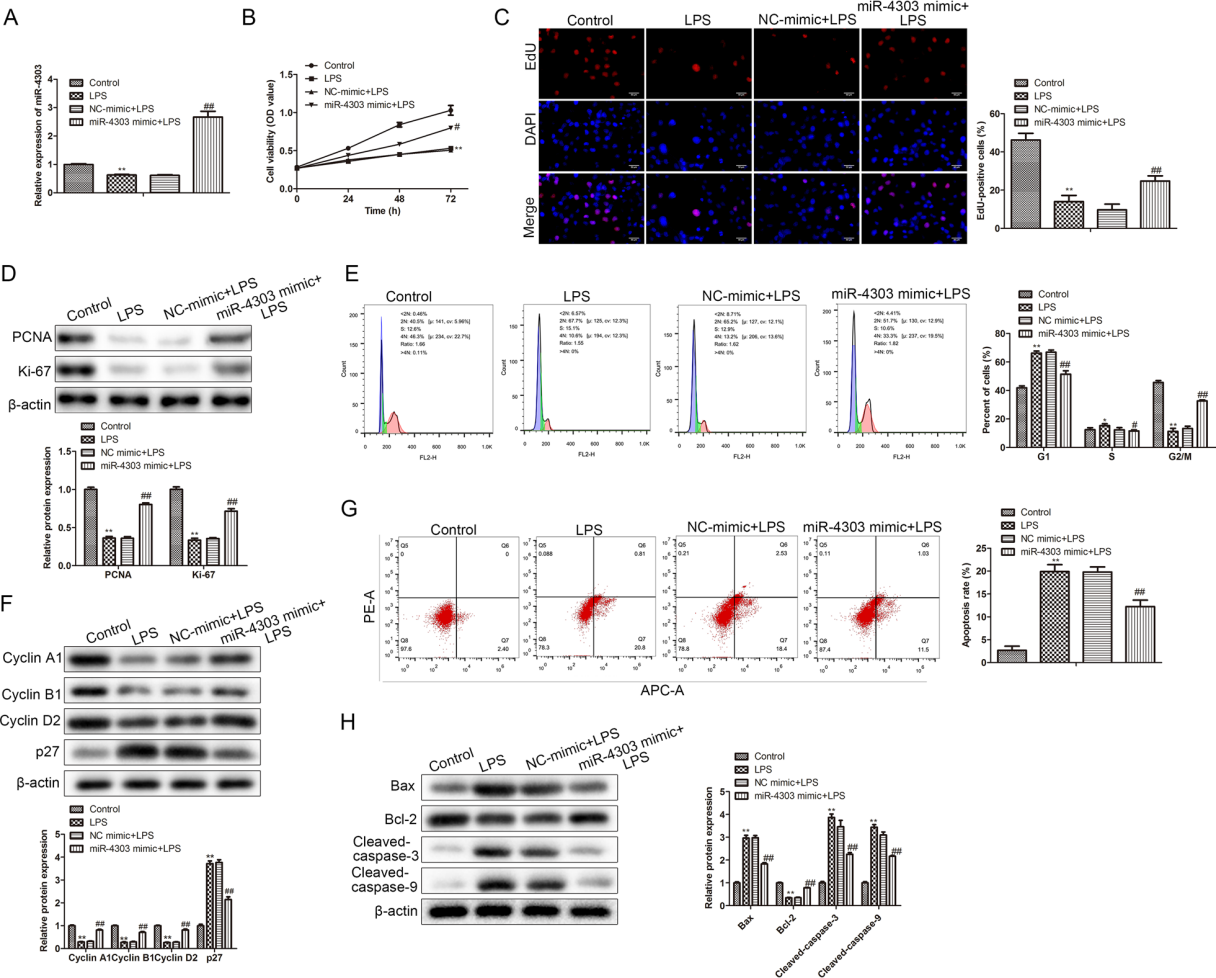
MiR-4303 overexpression rescues LPS-inhibited chondrocyte viability, proliferation and cell cycle and alleviates LPS-induced chondrocyte apoptosis. Chondrocytes were randomly divided into four groups, control group; chondrocytes were untreated; LPS group, 1 × 10^5^ chondrocytes were treated with 10 μL LPS (100 ng/mL); NC mimic + LPS group, 1 × 10^5^ chondrocytes were treated with 10 μL LPS (100 ng/mL) and 100 nM NC mimics; miR-4303 mimic + LPS group, 1 × 10^5^ chondrocytes were treated with 10 μL LPS (100 ng/mL) and 100 nM miR-4303 mimics. Untreated chondrocytes served as the negative control for LPS-treated chondrocytes; NC mimics served as the negative control for miR-4303 mimics. **A** RT-qPCR was performed to assess the levels of miR-4303. **B** CCK-8 assay was conducted to evaluate cell viability. **C** EdU assay was carried to measure cell proliferation. **D** Western blot was conducted to measure the levels of proliferation-related proteins, including PCNA and Ki-67. **E** FACs were performed to evaluate the distribution of chondrocytes in each cell cycle. **F** Western blot was conducted to measure the levels of cell cycle-related proteins, including CyclinA1, CyclinB1, CyclinD2 and p27. **G** FACs were conducted to assess chondrocytes apoptosis. **H** Western blot was performed to detect the levels of apoptosis-related proteins, including Bax, Bcl-2, Cleaved-casapase3 and Cleaved-casapase9. Tukey post-test was used for comparison between multiple groups. *P < 0.05, **P < 0.01 versus control group; ^#^*P* < 0.05, ^##^*P* < 0.01 versus NC mimic + LPS group. Error bars represented SD. Data represented three independent experiments

To further investigate the effect of miR-4303 on LPS-treated chondrocyte bioactivities, RT-qPCR was carried to assess the level of miR-4303 in chondrocytes. Findings indicated that LPS-inhibited miR-4303 expression compared with control, but miR-4303 overexpression relieved the inhibitory effect (*P* < 0.01; Fig. 2A). CCK-8 and EdU assays were performed to evaluate chondrocyte viability and proliferation. Results analysis showed that compared with control, LPS-inhibited chondrocyte viability and proliferation, whereas miR-4303 overexpression alleviated the inhibitory effect of LPS on chondrocytes (*P* < 0.01; Fig. 2B, C). Subsequently, EdU assay results were verified by Western blot. Compared with control group, the levels of proliferation-related proteins, including PCNA and Ki-67, were inhibited by LPS, while it was alleviated by miR-4303 overexpression (*P* < 0.01; Fig. 2D). Furthermore, FACs analysis showed that LPS causes G1 arrest in chondrocytes, miR-4303 overexpression promoted G1 to G2/M transition. Namely, miR-4303 overexpression accelerated chondrocytes cell cycle (*P* < 0.05; Fig. 2E). Western blot was carried out to further verify by checking cell cycle-related proteins. Cyclin A1, Cyclin B1, Cyclin D2, were inhibited by LPS, and antagonized p27 protein expression was up-regulated by LPS. Overexpressing miR-4303 restored these changes (*P* < 0.01; Fig. 2F). Ultimately, apoptosis analysis indicated that LPS promoted chondrocytes apoptosis, but miR-4303 antagonized the promoting effects (*P* < 0.01; Fig. 2G). Similar results were obtained by detecting pro-apoptotic protein, including Bax, Cleaved caspase-3 and Cleaved caspase-3 and anti-apoptotic protein Bcl-2 expression (*P* < 0.01; Fig. 2H). These findings revealed that MiR-4303 overexpression rescued the decrease in cell viability, cell cycle arrest and apoptosis induced by LPS.

### MiR-4303 relieves chondrocyte inflammation

**Fig. 3:**
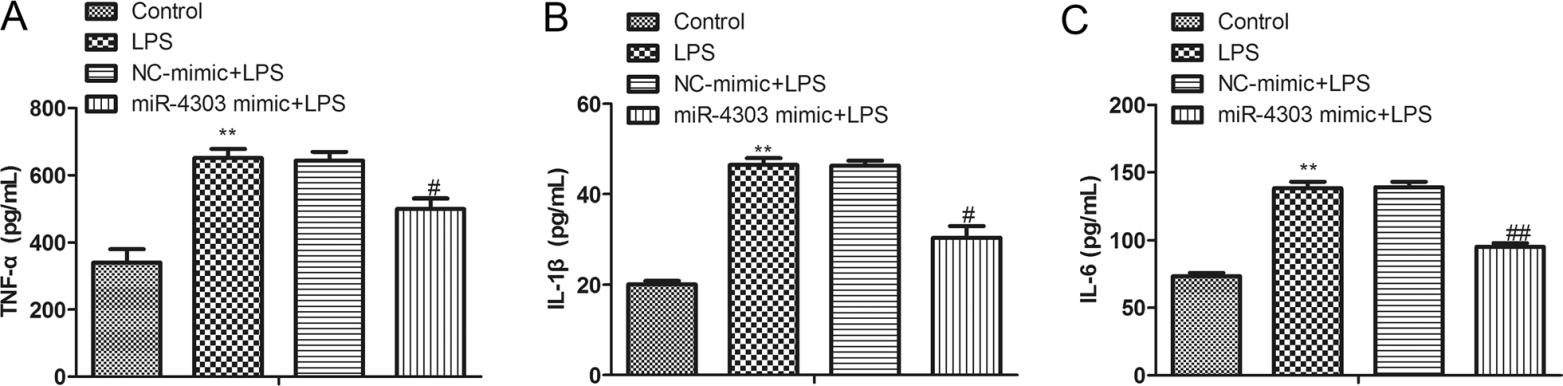
MiR-4303 overexpression inhibited the release of inflammatory factors from LPS-treated chondrocytes. Chondrocytes were randomly divided into four groups, control group; chondrocytes were untreated; LPS group, 1 × 10^5^ chondrocytes were treated with 10 μL LPS (100 ng/mL); NC mimic + LPS group, 1 × 10^5^ chondrocytes were treated with 10 μL LPS (100 ng/mL) and 100 nM NC mimics; miR-4303 mimic + LPS group, 1 × 10^5^ chondrocytes were treated with 10 μL LPS (100 ng/ml) and 100 nM miR-4303 mimics. Untreated chondrocytes served as the negative control for LPS-treated chondrocytes; NC mimics served as the negative control for miR-4303 mimics. **A** The levels of TNF-α in the supernatant of chondrocytes. **B** The levels of IL-β in the supernatant of chondrocytes. **C** The levels of IL-6 in the supernatant of chondrocytes. Tukey post-test was used for comparison between multiple groups. **P < 0.01 versus control group; ^#^*P* < 0.05, ^##^*P* < 0.01 versus NC mimic + LPS group. Error bars represented SD. Data represented three independent experiments

Osteoarthritis was accompanied by the release of a large number of inflammatory cytokines [20]. Therefore, an ELISA assay was established to explore the effect of miR-4303 on inflammatory factor release in LPS-induced chondrocytes. The result showed that LPS accelerated the secretion of TNF-α, IL-β and IL-6. Overexpressing miR-4303 antagonized the promoting effect (*P* < 0.05; Fig. 3A–C). These findings indicated that miR-4303 overexpression alleviated arthritis.

### MiR-4303 targets ASPN

**Fig. 4:**
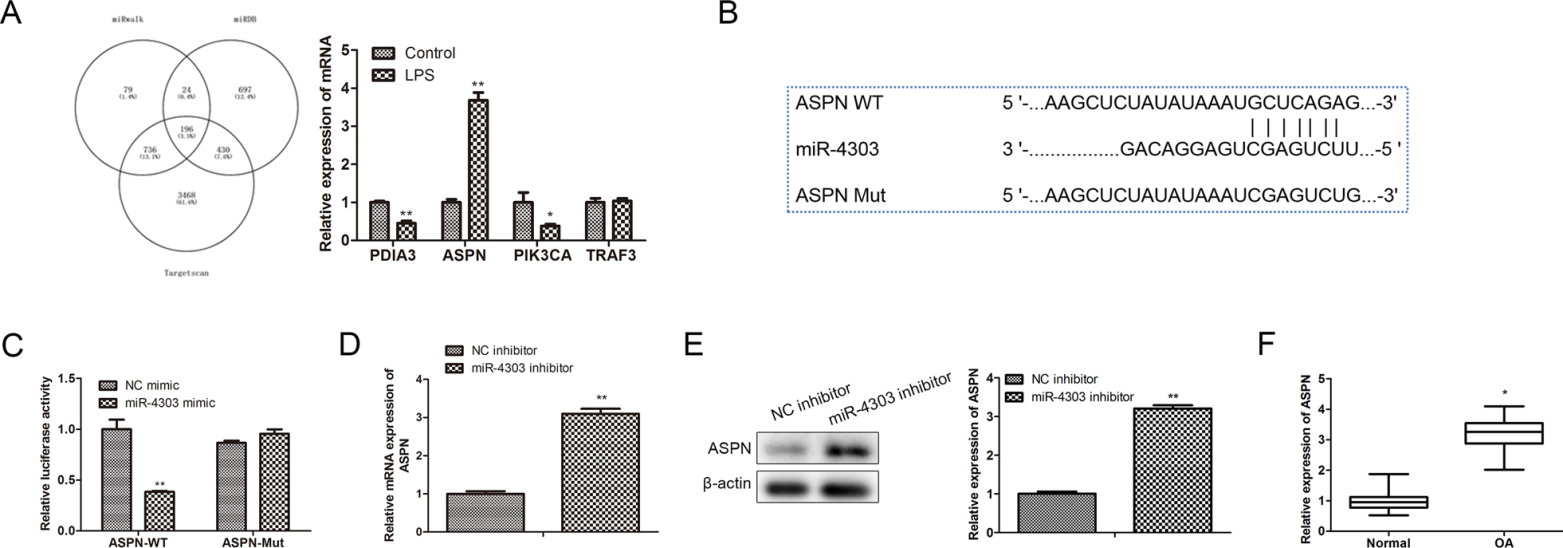
MiR-4303 targets ASPN. **A** Bioinformatics analysis was applied to predict the potential targets of miR-4303 downstream. Chondrocytes were treated with 10 μL LPS (100 ng/mL), untreated chondrocytes served as the negative control; RT-qPCR was performed to assess the mRNA levels of downstream effectors of miR-4303, including PDIA3, ASPN, PIK3CA and TRAF3. **B** The potential binding site between miR-4303 and ASPN. **C** The dual-luciferase reporter gene assay was conducted to confirm the direct binding relationship between miR-4303 and ASPN. MiR-4303 mimics were transfected into chondrocytes for miR-4303 overexpression, NC mimics served as the negative control for miR-4303 mimics. **D**, **E** RT-qPCR and western blot analyses were conducted to assess the levels of ASPN in chondrocytes following miR-4303 Inhibition. **F** The levels of ASPN in arthritic tissues, the corresponding normal tissues served as the negative control. Student's t test or one-way ANOVA was used to compare two groups, and Tukey post-test was used to compare multiple groups. *P < 0.05, *P* < 0.01 versus control group. Error bars represented SD. Data represented three independent experiments

MiRNAs participated in regulating disease progression by regulating the post-transcriptional translation of target genes [21]. Therefore, to determine the downstream regulator of miR-4303 in chondrocytes, bioinformatics analysis was adopted to predict the putative target gene of miR-4303. Venn diagram showed that a total of 196 downstream target genes of miR-4303 were detected, and RT-qPCR analysis indicated that compared with control, ASPN was significantly up-regulated in LPS-treated chondrocytes, instead of PDIA3, PIK3CA and TRAF3 (*P* < 0.05; Fig. 4A). Further analysis showed that the binding sites of miR-4303 and ASPN are presented in Fig. 4B. Subsequently, the dual-luciferase reporter gene assay indicated that miR-4303 mimics inhibited the luciferase intensity of the wild-type-ASPN reporter, but not the mutant-ASPN reporter (*P* < 0.01; Fig. 4C). Moreover, RT-qPCR and western blot analyses indicated that compared with control, miR-4303 inhibition up-regulated ASPN expression, and ASPN was significantly up-regulated in arthritic tissues (*P* < 0.05; Fig. 4D–F). These findings revealed that miR-4303 targeted ASPN in chondrocytes, ASPN played a positive role in chondrocyte inflammation.

### MiR-4303 relieves chondrocyte inflammation via targeting ASPN

**Fig. 5:**
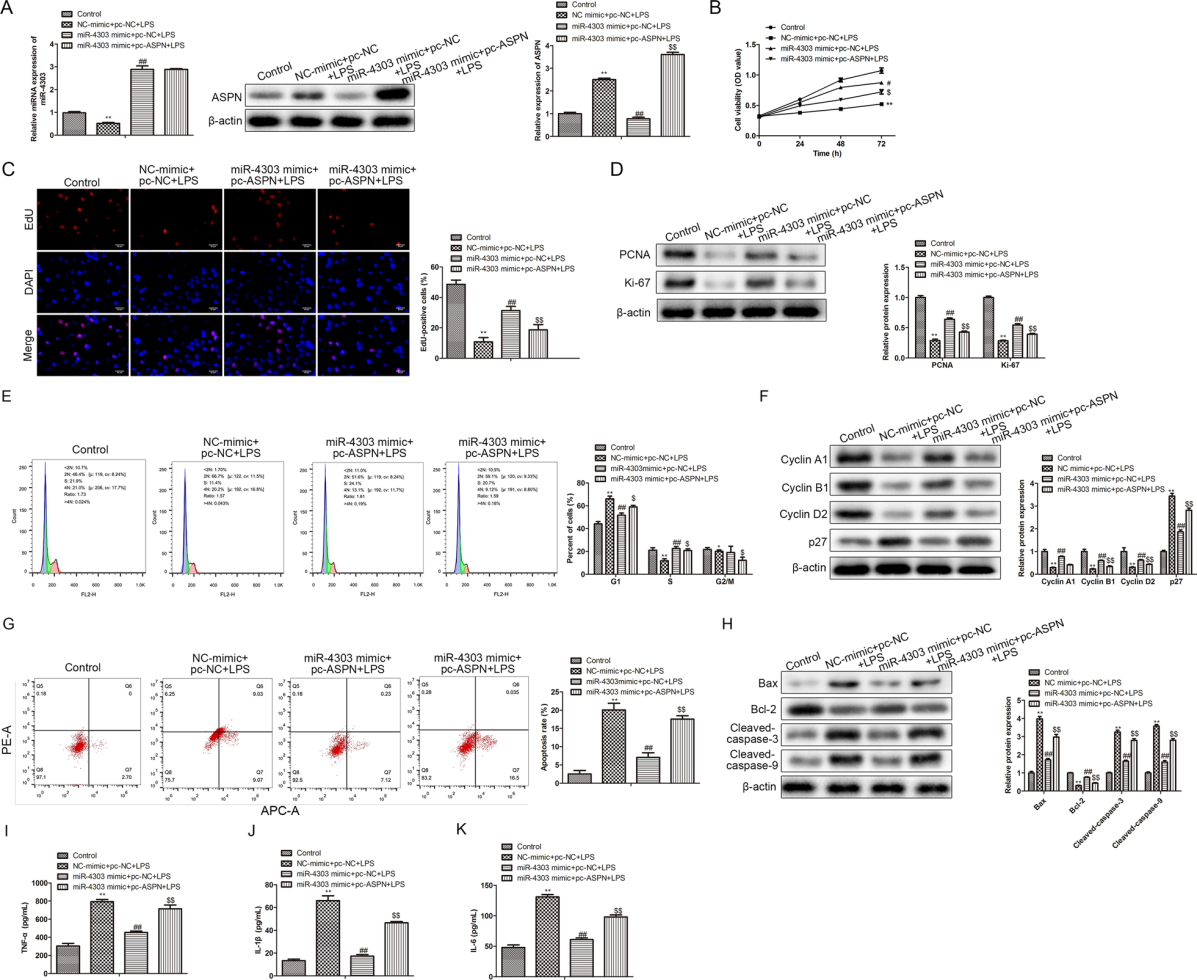
MiR-4303 relieves chondrocyte inflammation via targeting ASPN. Chondrocytes were randomly divided into four groups, control group; chondrocytes were untreated; NC mimic + pc-NC + LPS group, 1 × 10^5^ chondrocytes were treated with 10 μL LPS (100 ng/mL), 100 nM NC mimics were transfected into chondrocytes, and 500 ng pc-NC were transfected into chondrocytes; miR-4303 mimic + pc-NC + LPS group, 1 × 10^5^ chondrocytes were treated with 10 μL LPS (100 ng/ml), 100 nM miR-4303 mimics were transfected into chondrocytes, and 500 ng pc-NC were transfected into chondrocytes; miR-4303 mimic + pc-ASPN + LPS group, 1 × 10^5^ chondrocytes were treated with 10 μL LPS (100 ng/ml), 100 nM miR-4303 mimics were transfected into chondrocytes, and 500 ng pc-ASPN were transfected into chondrocytes. **A** RT-qPCR and Western blot were performed to assess miR-4303 and ASPN protein expression levels, respectively. **B** CCK-8 assay was performed to detect chondrocyte viability. **C** EdU assay was carried to evaluate chondrocyte proliferation. **D** Western blot was conducted to measure the levels of proliferation-associated proteins, including PCNA and Ki-67 in chondrocytes. **E** FACs were performed to evaluate the distribution of chondrocytes in each cell cycle. **F** Western blot was conducted to measure the levels of cell cycle-related proteins, including CyclinA1, CyclinB1, CyclinD2 and p27. G, FACs were conducted to assess chondrocytes apoptosis. H, Western blot was performed to detect the levels of apoptosis-related proteins, including Bax, Bcl-2, Cleaved-casapase3 and Cleaved-casapase9. I-K, ELISA was performed to detect the levels of TNF-α, IL-1βand IL-6 in the supernatant of chondrocytes. Tukey post-test was used for comparison between multiple groups. *P < 0.05, **P < 0.01 versus control group; ^#^*P* < 0.05, ^##^*P* < 0.01 versus NC mimic + pc-NC + LPS group; ^$^*P* < 0.05, ^$$^*P* < 0.01 versus miR4303 mimic + pc-NC + LPS group. Error bars represented SD. Data represented three independent experiments

Based on the above results, rescue assays were performed to demonstrate whether miR-4303 exerted its function in chondrocyte via targeting ASPN. RT-qPCR analysis indicated that miR-4303 mimic significantly increased the expression of miR-4303, while overexpression of ASPN did not affect its expression (*P* < 0.01; Fig. 5A, left). Western blot analysis showed that miR-4303 mimic significantly inhibited the expression of ASPN, on the basis of which overexpression of ASPN could supplement its expression. (*P* < 0.01; Fig. 5A, right). Subsequently, CCK-8 and EdU analyses depicted that miR-4303 mimic alleviated the cell viability inhibition induced by LPS, whereas such an impact was subsequently antagonized by ASPN overexpression (*P* < 0.05; Fig. 5B, C). A similar conclusion can be made from western blot assay (*P* < 0.01; Fig. 5D). Furthermore, FACs analysis depicted that the retardation of cell interphase was abolished by miR-4303 mimic and was further antagonized by ASPN overexpression (*P* < 0.05; Fig. 5E). These findings were confirmed by Western blot (*P* < 0.01; Fig. 5F). FACs analysis further indicated that LPS-induced chondrocyte apoptosis was abolished by miR-4303 overexpression (*P* < 0.01; Fig. 5G). Western blot verified that ASPN overexpression relieved the effect of miR-4303 overexpression LPS-induced chondrocyte (*P* < 0.01; Fig. 5H). Ultimately, ELISA analysis indicated that the levels of TNF-α, IL-1β and IL-6 in LPS-treated chondrocytes were inhibited by miR-4303 mimic, whereas the impact was relieved by ASPN overexpression (*P* < 0.01; Fig. 5I–K). These findings revealed that miR-4303 overexpression promoted LPS-reduced chondrocyte viability, proliferation and cell cycle, inhibits LPS-induced chondrocyte apoptosis and inflammatory cytokine release via targeting ASPN, indicating that miR-4303 relieved chondrocyte inflammation via targeting ASPN.

## Discussion

Osteoarthritis is a chronic joint disease with the highest incidence among the elderly. Its clinical symptoms include joint pain and dysfunction. In severe cases, it may contribute to disability and seriously affect people’s health [22]. Accelerating chondrocyte proliferation and reducing the release of inflammatory cytokines may be an effective strategy for treating osteoarthritis. Our findings illustrated that miR-4303 relieved chondrocyte inflammation via targeting ASPN, and miR-4303 served as a prognostic biomarker for chondrocyte inflammation. In addition, our findings provided novel prognostic biomarkers for predicting the progression and prognosis of osteoarthritis.

The primary role of miRNA is to regulate the post-transcriptional translation of genes to control development and tissue homeostasis. Although miRNA is consistently vital in cartilage homeostasis [23], miRNAs abnormal expression in chondrocytes will cause diseases, the most common of which is osteoarthritis. For example, miR-1271 overexpression accelerates osteoarthritis progression [24]. Indeed, many miRNAs relieved osteoarthritis by regulating chondrocyte proliferation and inhibiting the release of inflammatory cytokines. For example, miR-206 is regulated by MSC-derived exosomes to promote chondrocyte proliferation and inhibit chondrocyte apoptosis from slowing down disease progression. In addition, the release of inflammatory cytokines is also suppressed to a certain extent [14]. Our findings indicated that miR-4303 expression was significantly down-regulated in osteoarthritis tissues and LPS-induced chondrocytes. Attentively, 1 × 10^5^ chondrocytes were treated with LPS (100 ng/ml) to induce chondrocyte inflammation in *vitro*. This finding showed that miR-4303 played a negative role in osteoarthritis, which might be an effective therapeutic target. Subsequently, functional studies showed that miR-4303 overexpression rescued the decrease in cell viability, cell cycle arrest and promotion of apoptosis induced by LPS, which would be helpful for the recovery of osteoarthritis. What is more, ELISA analysis showed that miR-4303 overexpression inhibited the release of inflammatory factors from LPS-treated chondrocytes, suggesting that miR-4303 overexpression relieved chondrocyte inflammation. Based on the above studies, we believed that miR-4303 overexpression relieved chondrocyte inflammation and inhibited osteoarthritis progression.

Analysis of genetic polymorphism of the ASPN gene shows that ASPN is closely related to various bone and joint diseases [14]. Our findings showed that ASPN was significantly up-regulated in osteoarthritis, indicating that ASPN played a positive role in osteoarthritis progression. Increasing evidence indicates that ASPN is regulated by a variety of miRNAs and participates in disease progression. For example, miR-101 inhibits colorectal cancer through targeted regulation of ASPN [25]. Our researches showed that miR-4303 targeted ASPN, and negatively regulate ASPN expression. Further functional research analysis showed that overexpression of ASPN could partially reverse the effect of miR-4303 on chondrocytes induced by LPS, suggesting that miR-4303 relieved chondrocyte inflammation via targeting ASPN.

In summary, our findings illuminated that miR-4303 was down-regulated both in vivo and in vitro; miR-4303 overexpression rescued LPS-inhibited chondrocyte viability, cell cycle arrest and alleviated apoptosis and the release of inflammatory factors from LPS-treated chondrocytes; miR-4303 targeted ASPN and relieved chondrocyte inflammation via targeting ASPN. In conclusion, miR-4303 serving as a prognostic biomarker for chondrocyte inflammation, relieving chondrocyte inflammation via targeting ASPN. Thus, our findings might provide novel prognostic biomarkers for predicting the progression and prognosis of osteoarthritis.

## Data Availability

The raw data supporting the conclusions of this manuscript will be made available by the authors, without undue reservation, to any qualified researcher.
